# *Angiostrongylus vasorum* and *Aelurostrongylus abstrusus*: Neglected and underestimated parasites in South America

**DOI:** 10.1186/s13071-018-2765-0

**Published:** 2018-03-27

**Authors:** Felipe Penagos-Tabares, Malin K. Lange, Jenny J. Chaparro-Gutiérrez, Anja Taubert, Carlos Hermosilla

**Affiliations:** 1 Institute of Parasitology, Justus Liebig University Giessen, 35392 Giessen, Germany; 2 CIBAV Research Group, Veterinary Medicine School, University of Antioquia, 050034 Medellin, Colombia

**Keywords:** *Angiostrongylus vasorum*, *Aelurostrongylus abstrusus*, Gastropod-borne diseases, Lungworms, Metastrongyloidea

## Abstract

The gastropod-borne nematodes *Angiostrongylus vasorum* and *Aelurostrongylus abstrusus* are global causes of cardio/pulmonary diseases in dogs and cats. In the last decade, the number of reports on canine and feline lungworms has increased in several areas of Europe and North America. The unspecific clinical signs and prolonged course of these diseases often renders diagnosis challenging. Both infections are considered as emerging and underestimated causes of disease in domestic pets. In South America, little information is available on these diseases, apart from occasional reports proving the principle presence of *A. vasorum* and *A. abstrusus*. Thus, the purpose of this review is to summarize reports on infections in both domestic and wildlife animals in South America and to increase the awareness on gastropod-borne metastrongyloid parasites, which also include important zoonotic species, such as *A. cantonensis* and *A. costaricensis.* This review highlights the usefulness of diagnostic tools, such as the Baermann funnel technique, serology and PCR, and proposes to include these routinely on cases with clinical suspicion for lungworm infections. Future national epidemiological surveys are recommended to be conducted to gain a deeper insight into the actual epidemiological situation of gastropod-borne parasitoses in South America.

## Background

The gastropod-borne metastrongyloid parasites *Angiostrongylus vasorum* and *Aelurostrongylus abstrusus* are known to affect the cardiopulmonary system of canids and the respiratory tract of felids, respectively [1]. These potentially pathogenic parasites have recently gained attention from the veterinary community due to their spread beyond the borders of known endemic areas, mainly in European countries as well as in North America [1–11]. Although they have been reported in both, domestic and wild canids/felids in different South American countries (see Table 1) [12–16], suggesting an endemic presence of both parasites in these regions, only a few epidemiological surveys haven been conducted on these parasites in the past decades. This indicates a neglected and underestimated status of these parasitoses not only by the Latin American veterinary but also by the parasitology community. Thus, more epidemiological research is required to obtain actual, consistent and detailed data on their epidemiology and actual disease occurrence and on the impact of canine angiostrongylosis and feline aelurostrongylosis on domestic and wild canid/felid populations in South America, as already performed in Europe [2, 3].

**Table 1:** Reports on *Angiostrongylus vasorum* infections in definitive hosts in South America

Definitive host species	Geographical location	No. of cases	Reference
*Cerdocyon thous*	Gioás and Angra Dos Reis, Rio de Janeiro, Brazil	1	Travassos, 1927 [49]
*Cerdocyon thous/Canis familiaris*	Brazil	na	Dougherty, 1946 [141]
*Cerdocyon thous/ Canis familiaris*	Colombia and Rio Grande do Sul, Brazil	1	Gonçalves, 1961 [13]
*Canis familiaris*	Rio de Janeiro, Brazil	na	Langenegger et al., 1962 [54]
*Canis familiaris*	Rio de Janeiro, Brazil	na	Grisi, 1971 [50]
*Canis familiaris*	Paraná, Brazil	na	Giovannoni et al, 1985 [55]
*Canis familiaris*	Minas Gerais, Brazil	na	dos Santos et al., 1985 [56]
*Canis familiaris*	Argentina	na	Venturini & Borel, 1991 [58]
*Lycalopex* (syn*. Dusicyon*) *vetulus*	Minas Gerais, Brazil	4/8	Lima et al., 1994 [12]
*Lycalopex gymnocercus*	Bolivian Chaco	1/10	Fiorello et al., 2006 [82]
*Cerdocyon thous*	Minas Gerais, Brazil	3/6	Duarte et al., 2007. [57]
*Nasua nasua*	Paraná, Brazil	1	Vieira et al., 2008 [52]
*Eira barbara*	Mato Grosso do Sul, Brazil	1	Vieira et al., 2008 [52]
*Cerdocyon thous*	Pereira, Colombia	1	Varela-Arias et al., 2014 [59]
*Cerdocyon thous*	Federal District, Midwestern Brazil	1	Ferreira-Júnior et al., 2017 [142]
*Cerdocyon thous*	Minas Gerais, Brazil	2	Viera et al., 2017 [143]

*Abbreviation*: na, not applicable

It is well known that both canine angiostrongylosis and feline aelurostrongylosis can lead to certain diagnostic challenges due to the intermittent excretion of first-stage larvae (L1), the high variability of clinical signs and the frequently occurring chronic and subtle course of infections [2, 17, 18]. In addition, a reliable definitive diagnosis based on clinical-pathological, serological, molecular or coprological approaches is challenging, since all conventional diagnostic methods may fail due to certain deficiencies and limitations of each diagnostic method [2]. The best diagnostic tool for the detection of *A. vasorum* or *A. abstrusus* first larvae in faeces is still represented by the Baermann funnel migration technique, which is unfortunately rarely utilized in small animal veterinary clinics of South America [19] even though it is a cheap and easy diagnostic method [20]. As a consequence, only fragmentary information on the true geographical distribution and actual prevalence of these nematodes is available in South and Central America [19, 21].

**Fig. 1:**
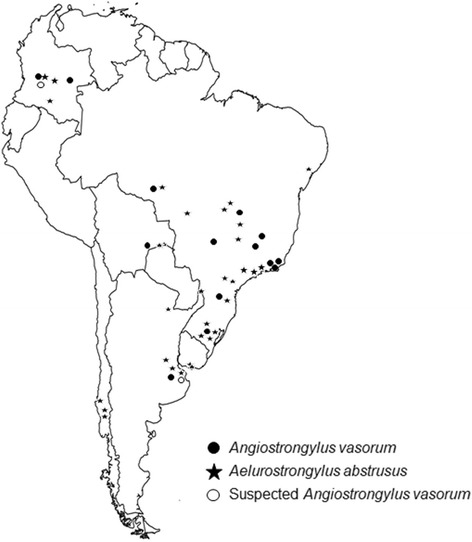
Reports of natural occurring infections of *Angiostrongylus vasorum* and *Aelurostrongylus abstrusus* in South America

Overall, some cases of both canine angiostrongylosis and feline aelurostrongylosis were described in several regions of South America (Fig. 1). Furthermore, some reports exist on a specific intermediate host species, the highly invasive terrestrial African giant snail *Achatina fulica* [22–24]*.* Between 1988 and 1989 this invasive neozoan snail species, originating from East Africa, was introduced to South America (especially to Brazil) for the commercial heliculture industry (snail farming) for human consumption [25]. Since then, this species rapidly spread throughout several South American countries, including Argentina, Colombia, Ecuador, Paraguay, Peru and Venezuela [26]. More importantly, *A. fulica* is considered as the most harmful invading terrestrial snail species on Earth [27, 28] and therefore might contribute to the spread of the here reviewed lungworms and other closely related anthropozoonotic metastrongyloid parasites, such as *Angiostrongylus costaricensis* and *A. cantonensis* as already reported for the Americas [24, 25].

The geographical expansion of lungworm infections, which was reported for several countries [4, 10, 29, 30] throughout the last decades, may rely on climate changes influencing the intermediate host-parasite relationship. In addition, international travelling activities of domestic dogs/cats throughout South America significantly increased in the last years, posing an enhanced risk of parasite import and transmission to previously non-endemic areas as already reported for Europe [20]. Furthermore, the population dynamics of gastropods are strongly affected by a variety of abiotic factors such as temperature and humidity [31]. Taking into account that terrestrial snails/slugs play a crucial role in the life-cycle of *A. vasorum* and *A. abstrusus* [21], an improvement of environmental conditions for gastropods will promote an increased occurrence of aelurostrongylosis and angiostrongylosis in new geographical areas as postulated elsewhere [24]. In addition, wildlife reservoirs, such as red foxes (*Vulpes vulpes*), crab-eating foxes (*Cerdocyon thous*), coyotes (*Canis latrans*), bush dogs (*Speothos venaticus*) and a wide spectrum of wild felid species in South America, request further consideration for the better understanding of the epidemiology. As such, the spread of foxes from sylvatic into suburban/urban areas is a well-known phenomenon in many geographical areas [32]. This ‘fox urbanization’ can obviously contribute to the import of infectious parasitic stages to domestic dog habitats as recently demonstrated for central Europe [33]. Besides, also wild felids might become urbanized in South American tropical/subtropical cities with vast forested park areas, thus possibly contributing to the spread of feline aelurostrongylosis.

The purpose of this review is to summarize currently available data on the epidemiological situation of *A. vasorum* and *A. abstrusus* infections in dogs, cats and wildlife animals in South America. Given that these parasites indeed occur in several countries of this continent and therefore should be considered as differential diagnoses in cases of canine cardiopulmonary/feline respiratory disease, the Baermann funnel technique should be included in routine diagnosis. Therefore, important informative aspects, such as the principles of the Baermann funnel technique, larval morphological characteristics and clinical signs are reviewed to encourage veterinarian surgeons and parasitologists to start investigations on these neglected diseases. Finally, novel diagnostic tools, such as serological and molecular approaches, are described briefly to stimulate future large-scale epidemiological surveys on lungworm infections, not only in domestic but also in wildlife animals of South America.

## Canine angiostrongylosis in South America

*Angiostrongylus vasorum* (Baillet, 1866), also known as ‘French heartworm’, is a parasite of domestic dogs and wild species of the family Canidae, including foxes [34], coyotes [21] and wolves [35, 36] amongst others. Furthermore, this nematode has been reported to occur in other closely related carnivore species, such as mustelids and the red panda (*Ailurus fulgens*) [21]. Experimentally, the Nile rat (*Arvicanthis niloticus*) proved a suitable final host [37]. *Angiostrongylus vasorum* shows a rather patchy geographical distribution worldwide [4, 6, 12, 38–40] and its geographical spread and infection incidence are considered as increasing in the recent years [2, 41]. A broad range of terrestrial snail and slug intermediate host species (e.g. *Arion ater*, *A. rufus*, *A. lusitanicus*, *A.fulica*, *A. distinctus*, *A. hortensis*, *Limax maximus*, *Helix aspersa* and *Tandonia sowerbyi*) [42–45] are infected by L1 either by ingestion while feeding on faeces or by active penetration through the gastropod epidermis [34]. Merely experimentally, the common frog (*Rana temporaria*) and the domestic chicken (*Gallus gallus*) were demonstrated as paratenic hosts for *A. vasorum* [46, 47]*.* Within the definitive host, adult nematodes mainly reside in the right heart and pulmonary arteries. Adult fertilized females produce eggs which embryonate and hatch within the pulmonary capillaries where L1 penetrate into the alveoli [2], migrate into the trachea, larynx, buccal cavity, are swallowed and finally shed through faeces into the environment.

Canine angiostrongylosis ranges from subclinical cases to severe cardiopulmonary and neurological disorders or coagulopathies besides inducing nonspecific clinical signs [48]. Even fatal infections are frequently reported [48]. Historically, *Angiostrongylus*-like nematodes in dogs and foxes have been denominated as *A. vasorum* in Europe and as *Haemostrongylus raillieti*, *Angiostrongylus raillieti* or *Angiocaulus raillieti* in Brazil [49–51]. Based on morphological and molecular characteristics, a recent taxonomic revision proposed to amalgamate all these species into one single parasite species, namely *A. vasorum* [41, 51]. However, genetic variations of European and Brazilian *A. vasorum* isolates suggested these isolates as separate cryptic species [41] but further investigations are needed to clarify the final taxonomy. Nonetheless, solving this taxonomic question requires a much larger sample size and the inclusion of additional isolates from different South American countries into genomic analyses, as previously suggested [41].

In Brazil, *A. raillieti* (syn. of *A. vasorum*) was reported in South American coati (*Nasua nasua*) and another *Angiostrongylus* sp. was described in the Tayra (*Eira barbara*)*.* In both wild mammal species, adult parasites were found in the lungs, heart and pulmonary arteries [51, 52]*.* Based on the specific cardiopulmonary localization and the historical confusion on the nomenclature, we here refer to these reported parasites as *A. vasorum.* Future research on these and other wildlife species is therefore mandatory to elucidate the natural definitive host spectrum of *A. vasorum* in a geographical region to be considered as mega-biodiverse as it is the South America subcontinent [53].

As the first South American report, *A. vasorum* was found in the right ventricle and pulmonary arteries of the crab-eating fox *C. thous* (Linnaeus, 1766) in Rio de Janeiro, Brazil [49]. Thereafter, natural *A. vasorum* infections were reported to occur in domestic dogs in Rio Grande do Sul, Brazil and in the crab-eating fox in Colombia [13]. Later on, more cases of *A. vasorum*-infected crab-eating foxes were described in other Brazilian regions, such as Rio de Janeiro [54], Paraná [55] and Minas Gerais [56, 57]. Additionally, in Minas Gerais *A. vasorum* was found as parasitizing hoary foxes (*Dusicyon vetulus*) (Lund, 1842) (syn. *Pseudoalopex* or *Lycalopex vetulus*) [12]. As also reported for North American free-ranging red foxes (*V. vulpes*) [38], a rather high *A. vasorum* prevalence of up to 50% was detected in Brazilian crab-eating foxes [57] (Table 1). Overall, the sum of these data indicates a broad distribution of *A. vasorum* in wildlife fox populations in South America which will contribute to the parasite propagation and the sylvatic life-cycle maintenance as already reported for Europe [57].

Unfortunately, there also exist ambiguous reports on *A. vasorum* or *Filaroides osleri* canid infections in South America lacking proper morphological diagnosis [58, 59]. One of these reports came from Argentina and included the coprological diagnosis ‘lungworm larvae’ for two domestic dogs without further characterization [58]. Another report was from Colombia, where a lethal infection of a crab-eating fox was described and diagnosed as lungworm infection based on histopathological findings of the lungs showing parasitic structures which resembled metastrongyloid parasites [59]. Based on the uncertain diagnosis of these two studies, it seems that more detailed research and instructions for veterinarians, pathologists and parasitologists in South America are required. For more detailed data on biology, epidemiology, diagnostic techniques, clinical features as well as anthelminthic treatments the following reviews on canine angiostrongylosis are recommended: Koch & Willesen [21], Helm et al. [44], Schnyder et al. [18] and Traversa & Guglielmini [2].

## Feline aelurostrongylosis in South America

*Aelurostrongylus abstrusus* (Railliet, 1898) infections are distributed worldwide [2, 19]. This parasite represents one of the most important etiological parasitic agents of respiratory alterations in domestic and wild felids [60, 61]. Clinical manifestations of feline aelurostrongylosis range widely from subclinical to a variety of respiratory signs such as dyspnoea, open-mouthed abdominal breathing, coughing, wheezing, sneezing and mucopurulent nasal discharge. Especially in cases of high-dose infections this parasitosis might have a clinical significance [62]. Analogous to *A. vasorum*, *A. abstrusus* has an indirect life-cycle involving a variety of terrestrial gastropods as intermediate hosts (i.e. *A. lusitanicus*, *L. maximus* [45], *A. fulica* [24, 45], *H. aspersa* [63]). Additionally, paratenic hosts such as rodents, frogs, lizards, snakes or birds are known to be involved in parasite life-cycle [60, 64]. In contrast to *A. vasorum*, adult stages of *A. abstrusus* reside in the terminal respiratory bronchioles, alveolar ducts and pulmonary alveoli, where the females produce eggs, which embryonate and hatch within the pulmonary ducts and alveoli [62].

Regarding the presence of *A. abstrusus* infections in South America, there are reports in domestic and wildlife felids from Uruguay [65, 66], Argentina [67, 68], Brazil [15, 69–76], Chile [77–80], Colombia [14, 16, 81] and Bolivia [82] (see Table 2 and Fig. 1). The first report ever on an *A. abstrusus* infection in a cat from South America came from Uruguay in the year 1942 [65]. In 1953, Trein [83] reported 40 cases out of 102 analysed domestic cats which had been submitted to necropsy in Rio Grande do Sul, Brazil. Thereafter, a prevalence of 8.6% was estimated *via* necropsy in cats from Montevideo during the period 1958–1960 [84]. During the 1970s and 1980s, domestic feline aelurostrongylosis was reported in Chile [77–79], Brazil [70] and Argentina [68]. In the 1990s, more reports on feline aelurostrongylosis came from Argentina and Brazil [71, 85, 86].

**Table 2:** Reports on *Aelurostrongylus abstrusus* infections in definitive and intermediate hosts

	Geographical location	Prevalence/no. of cases	Reference
Definitive host species			
*Felis catus*	Montevideo, Uruguay	1 case	Bacigalupo et al., 1942 [65]
*Felis catus*	Rio Grande do Sul, Brazil	40/102	Trein, 1953 [83]
*Felis catus*	Rio de Janeiro, Brazil	na	Langenegger and Lanzieri, 1963 [69]
*Felis catus*	Montevideo, Uruguay	8.6%	Esteves et al., 1961 [84]
*Felis catus*	Chile	1 case	Gonzalez & Torres, 1971 [77]
*Felis catus*	São Paulo, Brazil	na	Campedelli-Filho, 1972 [70]
*Felis catus*	Valdivia, Chile	na	Torres et al., 1972 [78]
*Felis catus*	Sâo Paulo, Brazil	8.5%	Fenerich et al., 1975 [144]
*Felis catus*	Valdivia, Chile	na	Bonilla-Zepeda, 1980 [79]
*Felis catus*	La Plata, and Buenos Aires Argentina	24.3%, 30.0%	Idiart et al., 1986 [68]
*Felis catus*	Corrientes, Argentina	30%	Martinez et al., 1990 [85]
*Felis catus*	Rosario, Argentina	na	Schiaffi et al., 1995 [86]
*Felis catus*	Santa Maria, Brazil	na	Headley & Conrado, 1997 [71]
*Puma yagouaroundi Leopardus geoffroyi*	Mato Grosso do Sul, Brazil	na	Noronha et al., 2002 [99]
*Felis catus*	Bogota, Colombia	1 case	Salamanca, 2003 [14]
*Felis catus*	Uberlândia, Brazil	18%	Mundim et al., 2004 [72]
*Felis catus*	Santa Maria, Brazil	5.9–25% (mean 18.6%, 1987–1996)	Headley, 2005 [73]
*Felis catus*	Buenos Aires, Argentina	2.6%	Sommerfelt et al., 2006 [67]
*Leopardus pardalis*	Bolivian Chaco	5 cases	Fiorello et al., 2006 [82]
*Leopardus geoffroyi*		3 cases	
*F.catus domesticus*	Rio de Janeiro, Brazil	1 case	Ferreira et al., 2007 [88]
*Felis catus*	Quindío, Colombia	0.21% (1/121)	Echeverry et al., 2012 [16]
*Felis catus*	Cuiaba and Várzea Grande, Matto Grosso, Brazil	1.3%	Ramos et al., 2013 [74]
*Felis catus*	Montevideo, Uruguay	2/8	Castro et al., 2013 [66]
*Felis catus*	Río Bueno y La Unión, Provincia del Ranco, Chile	20/200	Oyarzún-Cadagán, 2013 [80]
*Felis catus*	Rio Grande do Sul, Brazil	29.5%	Ehlers et al., 2013 [87]
*Felis catus*	Buenos Aires, Argentina	35.3% (6/17)	Cardillo et al., 2014 [91]
*Leopardus wiedii*, *Leopardus tigrinus*	Natural park De Trê Barras, Três Barras, Brazil	38.1% , 35.7%	Kusma et al., 2015 [15]
*Leopardus colocolo*	Rio Grande do Sul, Brazil	1 case	Gressler et al., 2016 [75]
*Felis catus*	Rio Grande do Sul, Brazil	22/2036 (1998–2005)	Pereira et al., 2017 [76]
*Felis catus*	Caquetá, Colombia	1 case	Sanchez-Rojas et al., 2017 [81]
Intermediate host species			
*Achatina fulica*	Rio de Janeiro, Goiás, Espırito Santo, Mato grosso, Sergippe and São Paulo, Brazil	5.57% (217/3806)	Thiengo et al, 2008 [90]
*Achatina fulica*	São Paulo, Brazil	na	Ohlweiler et al., 2010 [145]
*Achatina fulica*	Puerto Iguazu, Argentina	2%	Valente et al., 2017 [24]
*Rumina decollate*	Buenos Aires, Argentina	80% (20/25)	Cardillo et al., 2014 [91]

*Abbreviations*: na, not applicable

The report on the highest altitude so far, referred to an incidentally diagnosed *A. abstrusus* infection in a cat from Bogota (Colombia) [14], which is located approximately 2600 meters above sea level (masl), proving the resilience of gastropod intermediate hosts. In 2012, another incidental case of feline aelurostrongylosis diagnosed *via* the Ritchie test came from Quindío (Colombia) during a parasitological survey in domestic cats, this region has an average altitude of 1458 masl, which supports the fact that *A. abstrusus* is adapted to South American mountainous zones [16]. It is worth noting that the Ritchie test only proves positive in cases of highly parasitized and larvae-shedding animals since it is not specific for the detection of L1 in faeces [14]. An epidemiological study on 50 feline necropsies from Uberlândia, Minas Gerais, Brazil, in 2004, revealed an *A. abstrusus* prevalence of 18% [72]. One year later, a retrospective study on *A. abstrusus* infections in domestic cats presented for routine necropsy during 1987–1996 at the Federal University of Santa Maria, Brazil, detected a prevalence of 5.9–25 % [73]. In 2006, 2.6% of stray cats from Buenos Aires, Argentina, were found positive for *A. abstrusus* using the faecal flotation technique [67]. The prevalence of *A. abstrusus* in cats from Porto Alegre, state of Rio Grande do Sul, Brazil remained equal to 29.5% (24/88) during 2008 and 2009 [87].

In a survey in the metropolitan area of Cuiabá, Mato Grosso, Midwestern Brazil, cats revealed a prevalence of *A. abstrusus* of 1.3%, diagnosed *via* necropsy [74]. A recent report came from Chile, where 10% of domestic cats from the cities Rio Bueno and La Union showed *A. abstrusus* infections *via* the Bearmann funnel technique [80]. Recently, *A. abstrusus* was found by necropsy in two out of eight investigated cats in Montevideo, Uruguay [66]. Additionally, a recent retrospective study during 1998–2015 identified 22 cats with *A. abstrusus* infections in Rio Grande do Sul, Brazil [76]. Finally, other case reports on infected domestic cats originated from Rio de Janeiro, Brazil (2007; [88]) and Caquetá, Colombia (2017; [81]).

In addition to domestic felines, *A. abstrusus* infections are also reported in several wildlife species acting as definitive hosts, such as jaguarondis (*Puma yagouaroundi*) and Geoffroy’s cat (*Leopardus geoffroyi*), [89], margay (*L. wiedii*), oncilla (*L. tigrinus*) [15] and Colo colo wildcats (*L. colocolo*) [75]. Moreover, infected gastropod intermediate hosts have been reported in Brazil [90] and Argentina [24, 91] (see Table 2). As suitable intermediate host in South America, the terrestrial snail *Rumina decollate* has been reported in addition to the highly invasive African giant snail *A. fulica.* Interestingly, a rather high *A. abstrusus* prevalence was reported in 80% of *R. decollate* [91].

All above mentioned reports show that *A. abstrusus* cycles in both, sylvatic and urban areas. Therefore, it must be considered as differential diagnosis in cases of feline respiratory disease and in the management and conservation programmes on threatened wild felids in various regions of South America. For more details on the biology, epidemiology, pathophysiology, clinic, diagnosis and treatment options of feline aelurostrongylosis we recommend the recently published reviews of Elsheikha et al. [61] and Traversa & Di Cesare [19].

## Diagnostic tools for the detection of *A. vasorum* and *A. abstrusus* infections

### Coprological diagnostics

All coprological diagnostic methods described here share the limitation that they can be performed no earlier than seven weeks after the infection due to the parasites’ prepatency [92].

**Fig. 2:**
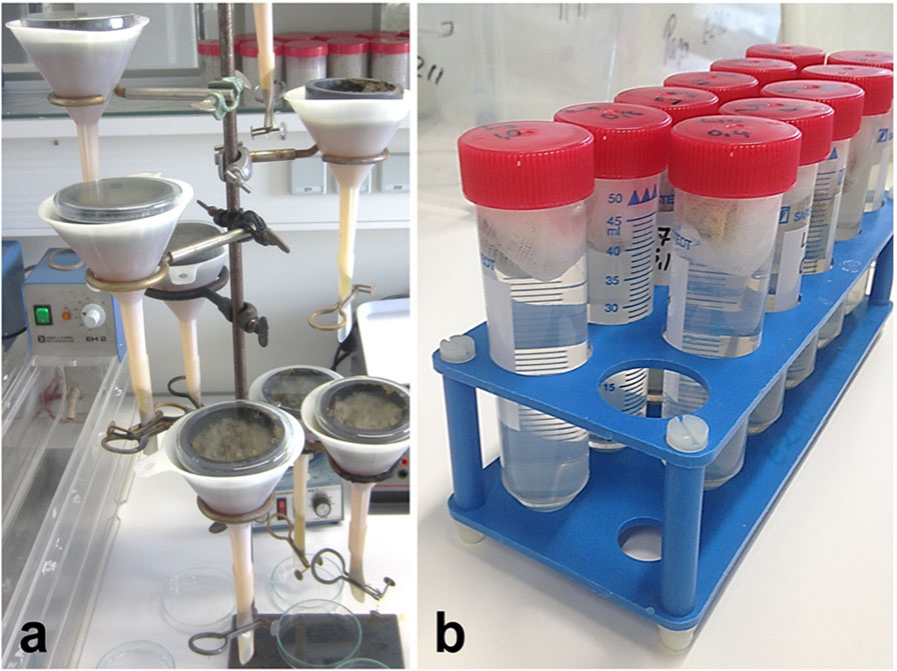
Illustration of modified Baermann funnel techniques. This technique is considered as the gold standard for the diagnosis of lungworm infections in cat and dog faeces. **a** A household funnel is combined with a plastic tube of 10 cm length and closed by a metal tubing clamp. The funnel is filled with handwarm tap-water. Then a wire mesh screen (9 cm diameter, 0.20–0.25 mm aperture) has to be set in the top of the funnel. **b** Modification by Conboy et al. [104]: instead of a funnel, 50 ml screw-top centrifuge tubes containing warm tap water are used. The faeces need to be placed in a double layer of cheesecloth, placed in the tube and the cap is screwed onto the tube catching a small part of the cheesecloth to keep it in place at the top at the tube. (Pictures taken by Malin K. Lange, Institute of Parasitology, Justus-Liebig-University Giessen)

In 1917, Baermann et al. [93] described a method to detect nematodes present in soil samples which was later on modified for lungworm larvae detection [94, 95]. This method is based on the hydrophilic and thermophilic behaviour of lungworm larvae [96]. Even though it is currently considered as a gold standard for the coprological diagnosis of feline and canine lungworm infections [21, 97], diagnosis may be hampered by the intermittent shedding of the larvae [98], a low viability of larvae [99], the seven week prepatency or scarce larval excretion in low-grade infections [100]. Therefore, the analysis of at least three samples from consecutive days is recommended by some authors [3, 101–103]. Since the Baermann funnel technique is an easy method that does not require specific equipment, it can be carried out in any veterinarian clinic (Fig. 2a). For small-sized samples, a modification of this technique was recently developed by Conboy et al. [104] using 50 ml screw top tubes as shown in Fig. 2b. Following a 12 h incubation, the larvae are here directly sedimented *via* centrifugation [104].

**Fig. 3:**
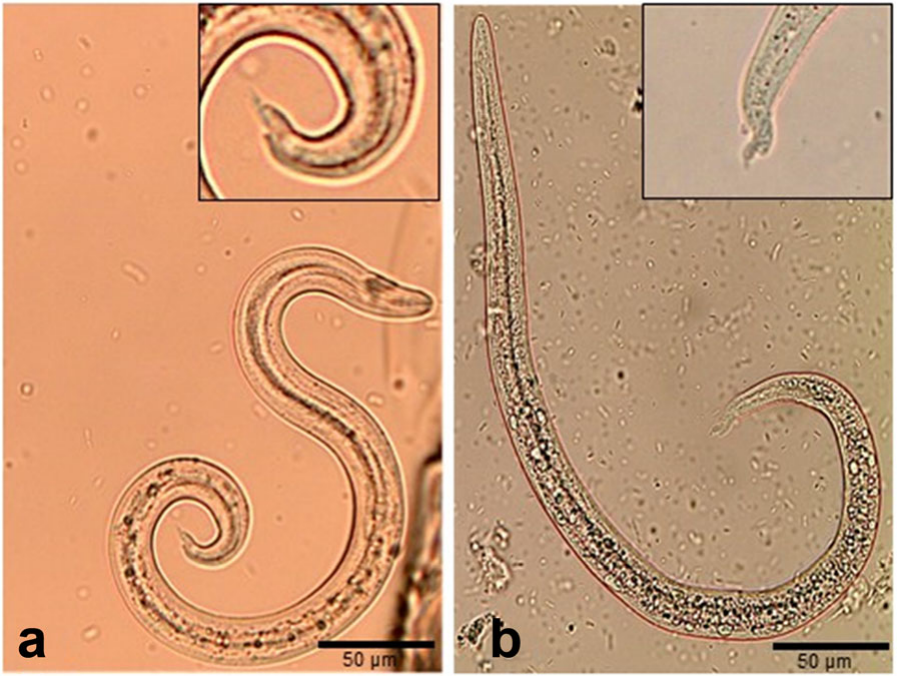
Morphological characteristics of first-stage larvae of *Angiostrongylus vasorum* and *Aelurostrongylus abstrusus*. **a** The first-stage larvae of *A. vasorum* possess a sinus wave curve formed tail end with a dorsal spine. **b** The first-stage larvae of *A. abstrusus* can be identified by the notched S-shaped tail. (Pictures taken by Malin K. Lange, Institute of Parasitology, Justus-Liebig-University Giessen)

Following the sedimentation step, L1 of metastrongyloid lungworms of domestic carnivores are differentiated microscopically *via* morphological characteristics which are mainly based on size (length, width, body/oesophagus ratio) and distinct tail morphology as reported elsewhere [63, 105–108]. A general morphological characteristic shared by all metastrongyloid lungworm L1 is the non-rhabditiform oesophagus, which forms 1/3–1/2 of the total larval length [4]. Considering the tail morphology, *A. abstrusus* L1 can be identified by its notched S-shaped tail (please see Fig. 3a), which is distinct from *A. vasorum* L1 possessing a sinus wave curve formed tail end with a dorsal spine (see Fig. 3b).

Another coproscopic technique for detection of lungworm larvae is the faecal smear [109], which is limited by a small sample size, includes no concentration step of parasitic stages, has a low sensitivity of 67% [2, 110] and is therefore not recommended as a routine method. The same applies for the zinc sulphate-based flotation method. In one survey, only 8/14 Baermann-positive fecal samples could be detected by this technique [111].

It is very important to emphasize that an accurate and definitive morphological identification of these parasites is a challenging process, which requires well-trained microscopists [2, 19]. So far, there are 181 species in the superfamily Metastrongylidae, many of which with a similar life-cycle and morphology [11]. Other less common parasitic larvae could be detected by the Baermann technique and their morphological and morphometrical key features should be considered since they could be confused with the two species on which this review is focused (see Table 3). In the case of canid samples, it is relevant to consider possible detection of *Crenosoma vulpis* and *Strongyloides stercolaris* [4]*. Strongyloides stercolaris* is spread worldwide [112], while *C. vulpis* is endemic in European and North American red fox (*Vulpes vulpes*) [30, 38] populations and rare in dogs [3]. There is a unique report in South America, specifically in Chile (2013), where 1% (2/200) of the evaluated canine faecal samples resulted positive by the Baermann funnel test [80].

**Table 3:** Canid and felid cardiopulmonary nematodes: differential characters of fisrt-stage larvae found by the Baermann funnel technique

Nematode (length × width) (μm)	Morphological keys	Final host	References
*Angiostrongylus vasorum* (310–400 × 14–16)	A small cup as a cephalic button emerges on the oral extremity	Canids	[1, 2, 4, 146, 147]
	Oesophagus non-rhabditiform, 1/3–1/2 the length of the larva		
	Tip with a dorsal spine and sinus wave curve		
*Crenosoma vulpis*^a^ (240–310 × 13)	Oesophagus non-rhabditiform, 1/3–1/2 the length of the larva	Canids	[4, 111, 148, 149]
	Tail, pointed and straight, without indentations and entirely pointed		
*Aulerostrongylus abstrusus* (300–415 × 18–19)	Anterior extremity slender, with a short/terminal oral opening leading into a narrow vestibule	Felids	[4, 63, 150, 151]
	Oesophagus non-rhabditiform, 1/3–1/2 the length of the larva		
	Tail S-shaped, with visible dorsal kink, distinct deep dorsal, ventral incisures, a terminal knob-like extremity		
*Troglostrongylus brevior*^b^ (300–357 × 16–19)	Anterior extremity clear and pointed, with a sub-terminal oral opening	Felids	[19, 113, 151–153]
	Oesophagus non-rhabditiform, 1/3–1/2 the length of the larva		
	Tail gradually tapered to dorsal incision, dividing the extremity into two appendices (shallow ventral one, slender dorsal one). S-shaped tail is not obvious, ending straight, gradually tapered		
*Troglostrongylus subcrenatus*^b^ (269–300 × 14–19)	Head pointed, oral opening subterminal (dorsal)	Felids	[19, 113, 153]
	Oesophagus non-rhabditiform, 1/3–1/2 the length of the larva		
	Tail gradually tapered to the extremity with deep dorsal incisure and shallower ventral incisure		
*Oslerus rostratus*^b^ (335–412 × 18–20)	Head rounded, with a central oral opening and a cylindrical buccal capsule	Felids	[1, 4, 151]
	Oesophagus non-rhabditiform, 1/3–1/2 the length of the larva		
	Tail slightly undulated, with a deep ventral notch (ending in minuscule spine) and a shallow dorsal notch		
*Angiostrongylus chabaudi*^b^ (307–420 × 14–16)	Cephalic extremity rounded, with a terminal buccal opening	Felids	[117, 146, 154, 155]
	Oesophagus non-rhabditiform, 1/3–1/2 the length of the larva		
	Caudal extremity with a small dorsal spine and notch, ending in a short sigmoid tail		
*Angiostrongylus felineus* ^c^	?	Felids	[118]
*Strongyloides stercoralis* (150–390 × 14–23)	Mouth with six lips, mouth-cavity rhabditiform, 1/20–1/21 of the total length of oesophagus	Felids and canids	[1, 4, 156]
	Oesophagus rhabditiform (corpus, isthmus, valvulated bulb), 1/4 of the total length of the larva		
	Pointed and straight tail		

^a^Europe and North America; unique report in South America (1% prevalence in dogs) in Chile [80]

^b^Not reported in South America

^c^Described in 2013 in *Puma yagouaroundi* from Brazil, first-stage larvae have been not described (Viera et al. [118])

Concerning the microscopical identification of felid coprological samples, it is mandatory to take into account the possible findings of *Troglostrongylus brevior*, *T. subcrenatus* [19, 113], *Oslerus rostratus* [19, 114–116], *Strongyloides stercolaris* [4], *Angiostrongylus chabaudi* [117], and *Angiostrongylus felineus* (recently discovered and described but only with adult stages in *Puma yagouaroundi* from Brazil; this is the reason why its L1 morphological description is lacking) [118]. *Troglostrongylus brevior*, *T. subcrenatus*, *A. chabaudi* and *O. rostratus* have been infrequently reported mainly in wild felids from Europe but not in South America [113, 116, 117]. Additionally, the existence of new metastrongyloid related species in a mega-biodiverse region such as South America could not be rejected and should be contemplated in future studies. Based on the prior observations, it is reasonable to consider the possibility of misdiagnosis in some reports of *A. vasorum* and *A. abstrusus* presented in this review, given that most of these studies were performed by microscopical identification, some of them many years ago, when surely the researchers were not aware of many above mentioned statements and species.

### Serological diagnostics

As mentioned before, the gold standard technique for the detection of *A. vasorum* is the Baermann larval migration test [21, 97]. However, this method is constrained by the intermittent shedding of the larvae [98], the seven week prepatency and scarce larval excretion in low-intensity infections [100]. To improve the efficiency and accuracy of *A. vasorum*-related diagnostics, new methods were developed. Thus, enzyme-linked immunosorbent assay (ELISA) tests have been designed to detect circulating *A. vasorum* antigens in serum samples with a specificity ranging between 94–100% and a sensitivity between 42.9–95.7% [99, 100, 119]. However, for some ELISAs non-specific reactions due to antigen-based cross-reactivity to other nematode infections were reported [92, 120]. Overall, *A. vasorum* antigen revealed as firstly detectable approximately five weeks after (experimental) infection and appeared to persists for a certain time period after elimination of the parasite [119]. Nevertheless, specific antigen detection may serve as a useful tool for treatment control, as previously proposed [99, 121] since antigen levels significantly decrease after treatment [119]. Thus, an absence of circulating antigens was observed in dogs treated with imidacloprid/moxidectin at 4 or 32 days post-infection (pi) and in dogs treated at 88–92 days pi, circulatory antigens decreased within 13–34 days [119]. Recently, a rapid *in situ* assay (Angio Detect™ Test, IDEXX Laboratories, Westbrook, Maine, USA) was merchandized for the serological detection of circulating *A. vasorum* antigens. This assay showed 100% sensitivity at 14 weeks pi and and with the earliest positive reaction at 9 weeks pi. [99]. When compared to the Baermann funnel assay, the Angio Detect^TM^ Test showed a sensitivity of 97.1% and a specificity of 98.9% [122]. Thus, this diagnostic test seems to be a useful diagnostic tool in a clinical setting.

Besides antigen detection, serological ELISA tests have been developed to detect specific antibodies raised against the parasite [122, 123]. However, antibody detection in a clinical context depends on the average life span of immunoglobulins and continued antigen stimulation [124]. In the early phase of infection specific antibodies can be detected while antigens are still not detectable [123]. *Angiostrongylus vasorum-*specific antibodies can be detected from 13 to 21 days after infection onwards persisting for up to nine weeks pi [125]. Thus, Cury et al. [97] detected *A. vasorum*-specific antibodies 14–28 days after experimental infection of dogs but humoral responses showed to be highly variable [126]. Serological tests for *A. vasorum*-specific antibody detection based on adult-, excretory/secretory (ES) antigens or L1 antigens showed a sensitivity of up to 85.7% and a specificity of 98.8% during prepatency [125]. However, Schucan et al. [125] found cross-reactions using adult somatic, adult ES antigens and L1 somatic antigen with sera of dogs infected with *C. vulpis*, *Dirofilaria immitis*, *D. repens* and *Eucoleus aerophilus*. When using monoclonal antibody-purified antigens, these cross-reactions were minimalized [125] and specificity was augmented [99, 119, 125, 127].

*Angiostrongylus vasorum*-specific antibodies can also be detected using the immunoblot (western blot) technique [97]. Although the sensitivity of western blots was higher than above mentioned antibody-ELISA [97], the former technique is only convenient for small sample sizes due to the large effort of this technique.

Both, antigen- and antibody-ELISAs were tested in a field study and compared to the Baermann funnel technique [123]. Thereby, the ELISAs principally confirmed Baermann-positive dogs and additionally detected non-patent infections [123]. As suggested by Schnyder et al. [127], the detection of parasite-specific antigen indicates an actual infection status, while parasite-specific antibodies merely reflect earlier parasite exposure. Consequently, such cases in which both, circulating *A. vasorum* antigens and specific antibodies are detected are assumed as active *A. vasorum* infections while exclusive antibody detection indicates infections that were acquired in the past [119, 125]. Nonetheless, it is important to note that antibody-seropositive dogs may also be free of parasites due to a self-curing process or treatment [125].

Regarding the serological diagnostics for *A. abstrusus*, these techniques are still in development and there are no commercially available serological tests for diagnosis of aelurostrongylosis [61]. Recently, an indirect fluorescent antibody test (IFAT) capable of detecting antibodies against *A. abstrusus* in sera from cats was developed and it showed to be promising in terms of sensitivity and specificity [128]. Furthermore, preliminary results suggest that the detection of antibodies using an ELISA might be a valuable tool for individual diagnosis and also for sero-epidemiological studies on feline aelurostrongylosis [61, 129].

Nonetheless, developing new diagnostic technologies with high sensitivity, specificity, availability and/or efficiency by means of improvement existing assays is necessary [61]. Presently, due to the lack of an optimal commercial serological diagnostic technique the Baermann method is recommended and could be employed for morphological detection of *A. abstrusus* infections which preferably should be confirmed by concurrent PCR. In the same way, larvae obtained from tracheal swabs or bronchoalveolar lavage could confirm the infection *via* PCR [2, 61]

### Polymerase chain reaction (PCR)-based diagnostics

Several studies used the PCR technique, mainly based on the second internal transcribed spacer (ITS2) region of ribosomal deoxyribonucleic acid (rDNA), in combination with sequencing of the amplified PCR product to confirm lungworm infections in dogs and wild carnivores [92, 130–135]. Using this molecular technique, different types of samples such as blood, faeces and mucosal smears or even intermediate hosts have successfully been used [132, 135]. However, the sensitivity and reliability of real-time PCR using ITS2 was dependent on the type of sample tested with blood being superior to faeces and pharyngeal or tracheal swabs regarding *A. vasorum* [92, 135, 136]. Houpin et al. [137] described a novel nested PCR- restriction fragment length (PCR-RFLP) [based on *18S* ribosomal ribonucleic acid (rRNA)] for the detection and identification of canine lungworms with a sensitivity of 69.5%. Copro-PCR-based analyses may also be useful in cases of Baermann funnel technique failure due to morphologically altered or less motile *A. vasorum* L1 [121]. However, false negative results in PCR-based analyses were reported depending on quality of the sample and amount of sample used for DNA extraction [92]. Therefore, PCR-based diagnostic techniques for detection of *A. vasorum* are considered less sensitive than ELISA and the Baermann funnel technique [92]. Nevertheless, PCR-based tools developed for the diagnosis of *A. abstrusus* infections showed a specificity of 100% and a sensitivity of ~97% [138]. In addition, an ITS2-based duplex PCR was developed to discriminate between *A. abstrusus* and *T. brevior* (a closely related lungworm parasite species) infections in a single cat [139]. Most recently, a triplex semi-nested PCR for the simultaneous detection of *A. abstrusus*, *T. brevior* and *A. chabaudi* (a rare cardiopulmonary nematode of wild felids) DNA was published [140].

Overall, since these novel molecular diagnostic tools have proven successful and effective for the diagnosis of canine angiostrongylosis and feline aelurostrongylosis thereby partially overcoming limitations of classical diagnostic methods, they may be useful to perform large-scale epidemiological surveys. However, the rather high costs of this molecular technique should also be taken into account, especially in poorer regions of South America.

## Conclusions

Considering the wide distribution of canine angiostrongylosis and feline aelurostrongylosis in South America, it is of great interest that small and wildlife practice clinicians consider these infections (and the less common species such as *C. vulpis*, *T. brevior*, *T. subrenatus*, *A. chabaudi*, *A. felineus* and *O. rostratus*) as differential diagnosis in the case of cardiopulmonary disorders. Here, the implementation of routinely applied tools of diagnostics, such as the Baermann funnel technique, parasitological dissection, PCR and/or serology is essential for South America regions since correct diagnosis of infections will significantly contribute to an improved knowledge on the current epidemiological situation of these neglected parasitoses. Phylogenetic studies are also pending to evaluate if *A. vasorum* from South America actually represents a distinct genotype or species. Epidemiological surveys in domestic and wild canid and felids as well as paratenic- and intermediate-hosts with an accurate molecular characterization are required. Additionally, the impact of climatic factors (e.g. altitude, temperature, annual precipitation, relative humidity and biogeographical region) on host-parasite and parasite-intermediate host interactions and the spread of these parasites into non-endemic regions are relevant topics to be considered in future investigations in one of the most biodiverse regions of the planet.

## Abbreviations

ELISA: enzyme-linked immunosorbent assay
ITS2: second internal transcribed spacer
L1: first-stage larvae
PCR: polymerase chain reaction
PCR-RFLP: polymerase chain reaction - restriction fragment length polymorphism
rDNA: ribosomal deoxyribonucleic acid
RNA: ribonucleic acid
rRNA: ribosomal ribonucleic acid
